# Controlling water-mediated interactions by designing self-assembled monolayer coatings

**DOI:** 10.1038/s41598-021-87708-8

**Published:** 2021-04-19

**Authors:** Hsieh Chen, S. Sherry Zhu

**Affiliations:** Aramco Services Company: Aramco Research Center-Boston, 400 Technology Square, Cambridge, MA 02139 USA

**Keywords:** Wetting, Surfaces, interfaces and thin films, Molecular dynamics, Nanoparticles

## Abstract

Engineered nanoparticles have been broadly used in biological and geological systems. Hydrophilic molecules such as polyols have been used as coatings on nanoparticle surfaces due to their good biocompatibility and solubility in saline water. However, polyol coatings can cause huge retention of nanoparticles when encountering mineral surfaces. Here, molecular dynamics simulations enlightened that the strong adhesion of hydrophilic coatings to mineral surfaces stemming from the partitioning of the hydroxy groups on the hydrophilic molecules to the well-defined bound hydration layers on the mineral surfaces. To mitigate the nanoparticle adhesion, we investigated introducing small percentages of omniphobic fluoroalkanes to form a bicomponent system of hydrophilic and fluoroalkanes, which greatly perturbed the hydration layers on mineral surfaces and resulted in nonstick surface coatings. Our results provide important insight for the design of tunable “stickiness” nanoparticle coatings in different mineralogies, such as applications in subsurface environments or targeted delivery in mineralized tissues.

## Introduction

Nanoparticle-mineral interactions are relevant in many biological, technological, and geological applications. In biomedicine, nanoparticles have been used as drug and gene carriers for bone regeneration and cancer therapy^1–4^, as well as MRI and CT contrast agents for musculoskeletal tissues^5,6^. In addition, natural or manmade nanoparticulates can play a key role in regulating or causing pathogenic biomineralization through heterogeneous nucleation and adhesion on mineral surfaces^7–9^. On the other hand, in geological and environmental applications, nanoparticles have been proposed for improving oil and gas recovery, flow diagnostics, CO_2_ sequestration, and environmental remediation, where the nanoparticles encounter extended mineral surfaces when traveling through subsurface formation matrices^10–16^.

Surface functionalization has been critical for the engineered nanoparticles to achieve the desired compatibility in the target environments. Hydrophilic coatings such as polyols and polysaccharides are attractive for their good biocompatibility and solubility in saline aqueous environments^17–19^. Nevertheless, polyols and polysaccharides may form extensive adsorption on mineral surfaces^20^. This property can be beneficial when using the polyol-functionalized nanoparticles as mineral flocculants or bone regeneration scaffolds^21–23^. However, the nonspecific adsorption can also be detrimental if the transport of nanoparticles through mineral interfaces without any retention is desired, such as using nanoparticles as subsurface interwell tracers^24–26^ or targeted drug delivery agents in specific bone locations^3,4^.

**Figure 1:**
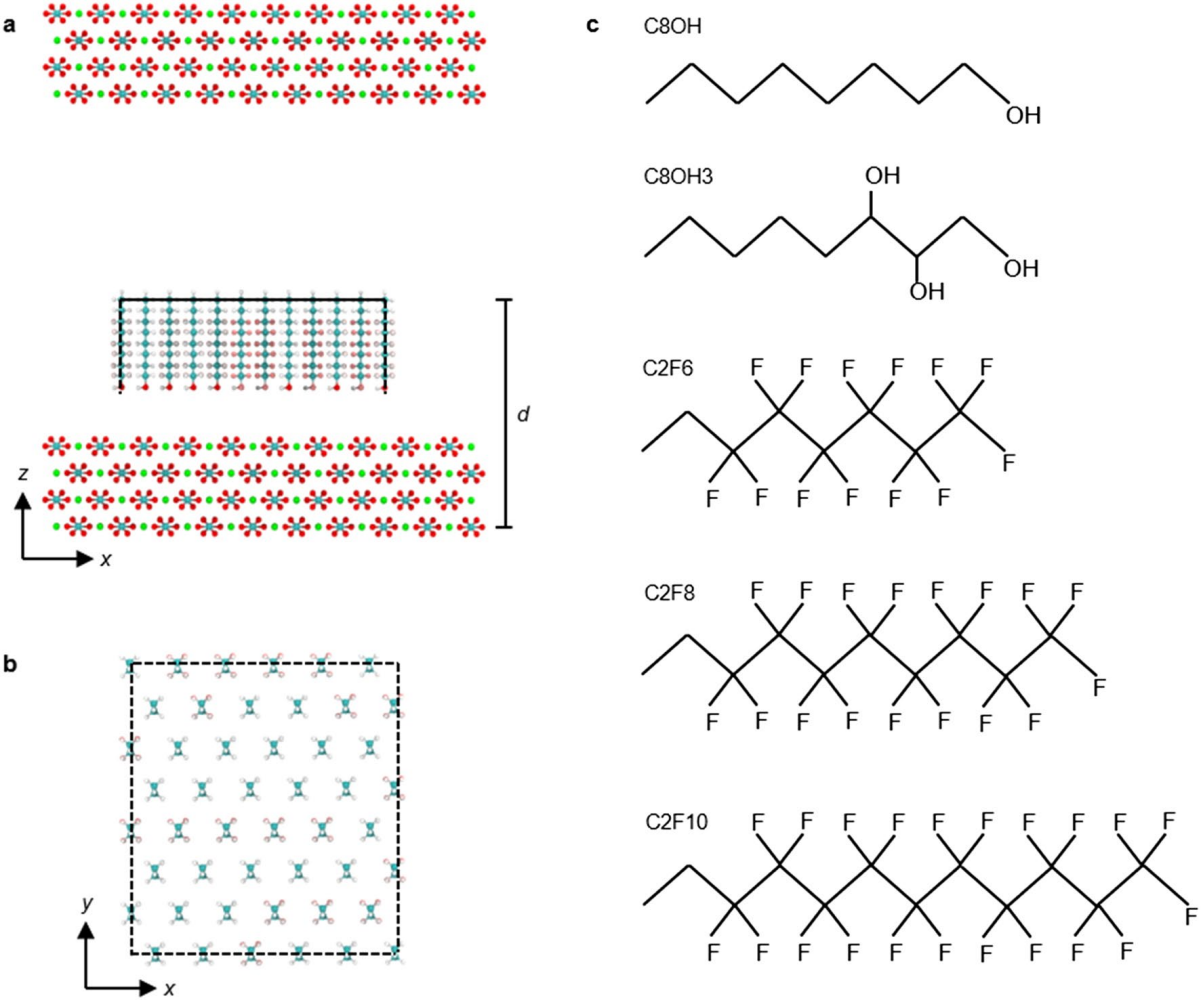
Simulation setup. (**a**) Side view of a bicomponent alkanol and fluoroalkane self-assembled monolayer (SAM) on a calcite (1 0 $\stackrel{-}{1}$ 4) surface separated with distance *d*. (**b**) Top view of the SAM with the alkanols and fluoroalkanes grafted onto a hexagonal lattice. The atom colors were: C = cyan, H = white, O = red, Ca = green, and F = pink. (**c**) Chemical structures and abbreviations of all the SAM molecules used in this study.

Despite the immediate relevance in many biomedical and geological applications, the detailed hydrophilic coating-mineral interactions have not been fully understood. In this paper, atomistic molecular dynamics (MD) simulations of hydrophilic self-assembled monolayer (SAM) interactions on calcite surfaces (Fig. 1) revealed an important mechanism that polyol-mineral interactions were mainly through the partitioning of the hydroxy groups on the polyols to the distinct hydration structures on the mineral surfaces. More importantly, based on the above physical picture, we tested a bicomponent system consisting of a small percentage of omniphobic fluoroalkane in the alkanols, which drastically perturbed the hydration structures on the mineral surfaces and resulted in a purely repulsive nonstick coating.

## Results

### Molecular origin of water-mediated self-assembled monomer adhesions on calcite surfaces

**Figure 2:**
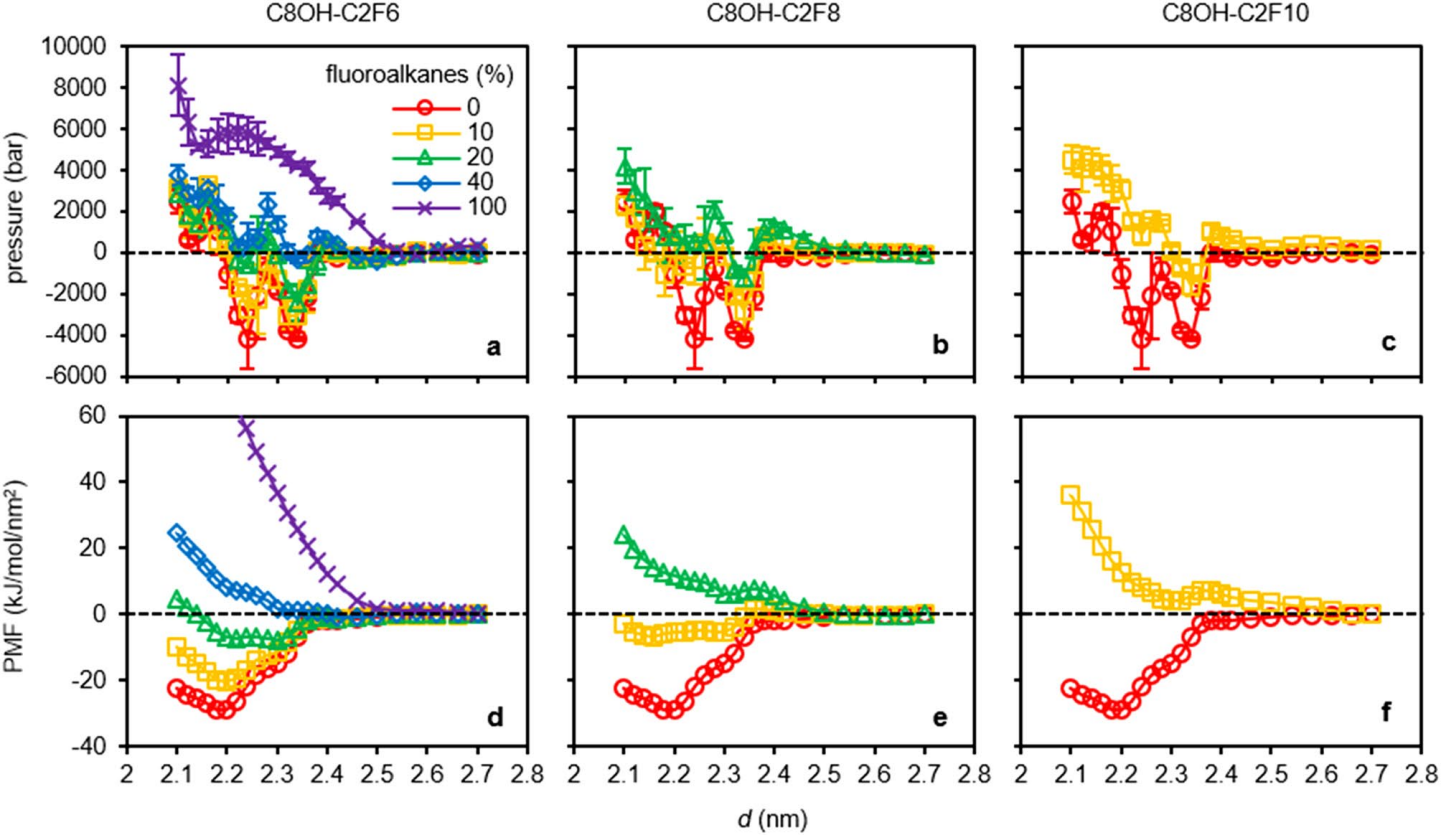
Pressure-distance curves and potential of mean forces (PMF) for the SAM-calcite interactions with different length and percentages of fluoroalkanes. (**a–c**) Pressure-distance curves and (**d–f**) PMF for the C8OH-C2F6 (**a** and **d**), C8OH-C2F8 (**b** and **e**), and C8OH-C2F10 SAM (**c** and **f**) interactions on calcite surfaces. The pressure-distance curves show two strong adhesion distances at *d* = 2.34 and 2.24 nm that correspond to the partitioning of the C8OH hydroxy groups to the second hydration layer (*d* = 2.34 nm) or second and first hydration layers (*d* = 2.24 nm) on calcite surfaces. The PMF show nonstick SAM-calcite interactions for 40% C2F6, 20% C2F8, or 10% C2F10 fluoroalkanes.

**Figure 3:**
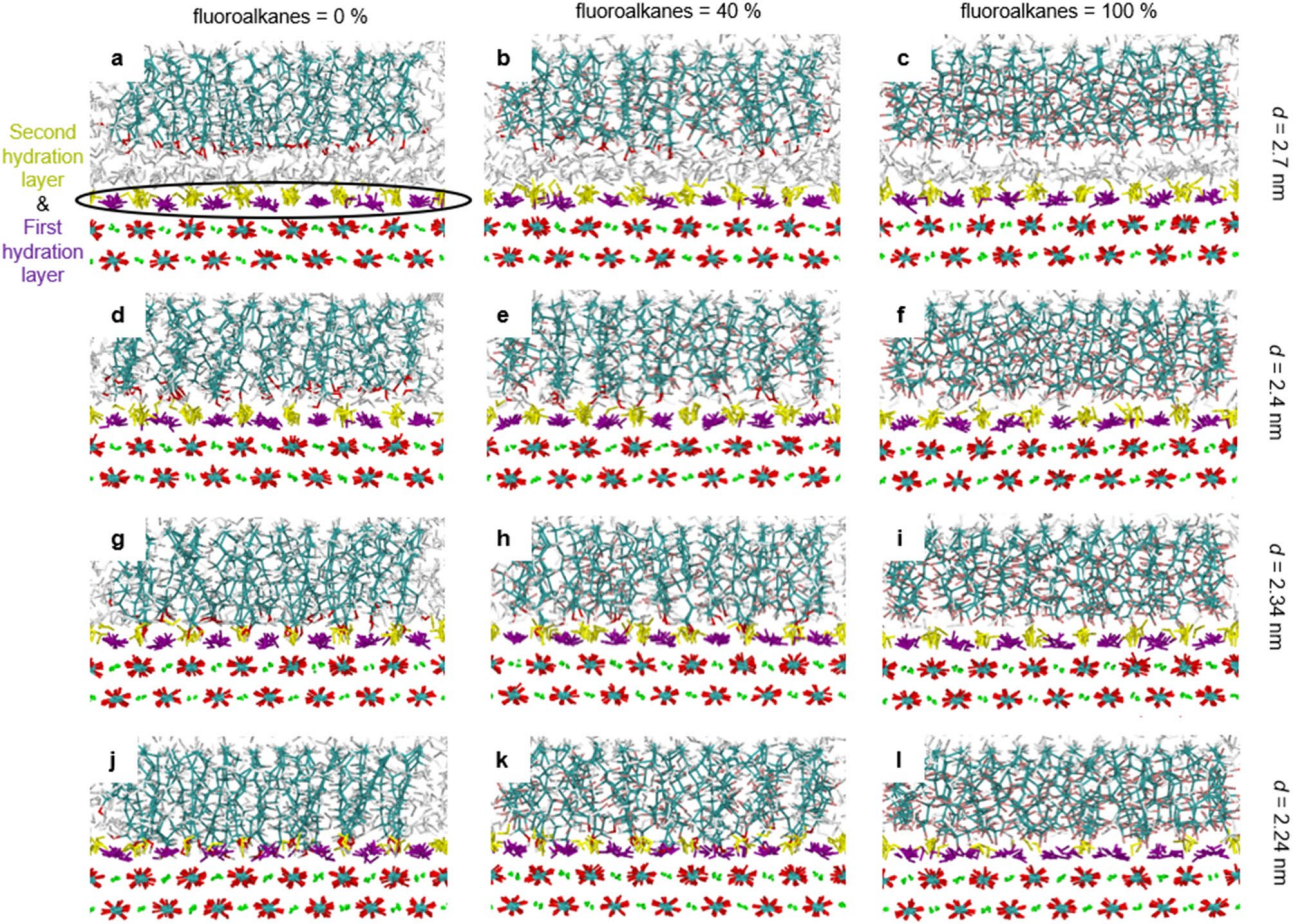
Simulation snapshots for different C8OH-C2F6 SAM-calcite distances with different percentages of fluoroalkanes. (**a–c**) *d* = 2.7 nm, (**d–f**) *d* = 2.4 nm, (**g–i**) *d* = 2.34 nm, and (**j–l**) *d* = 2.24 nm with 0% (**a**,**d**,**g**, and **j**), 40% (**b**,**e**,**h**, and **k**), and 100% (**c**,**f**,**i**, and **l**) fluoroalkanes. The atom colors of SAM and calcite were the same as in Fig. 1. The well-defined bound water molecules on the first and second hydration layers on calcite are colored with purple and dark yellow (see circled in **a**), respectively, and the rest of the water molecules are colored with gray. At *d* = 2.34 nm, the hydroxy groups on C8OH can participate in the second hydration layer (**g** and **h**). At *d* = 2.24 nm, the hydroxy groups on C8OH can participate in both the first and second hydration layers (**j** and **k**). In contrast, the fluoroalkanes cannot participate in the hydration layers (**h**,**i**,**k**, and **l**).

**Figure 4:**
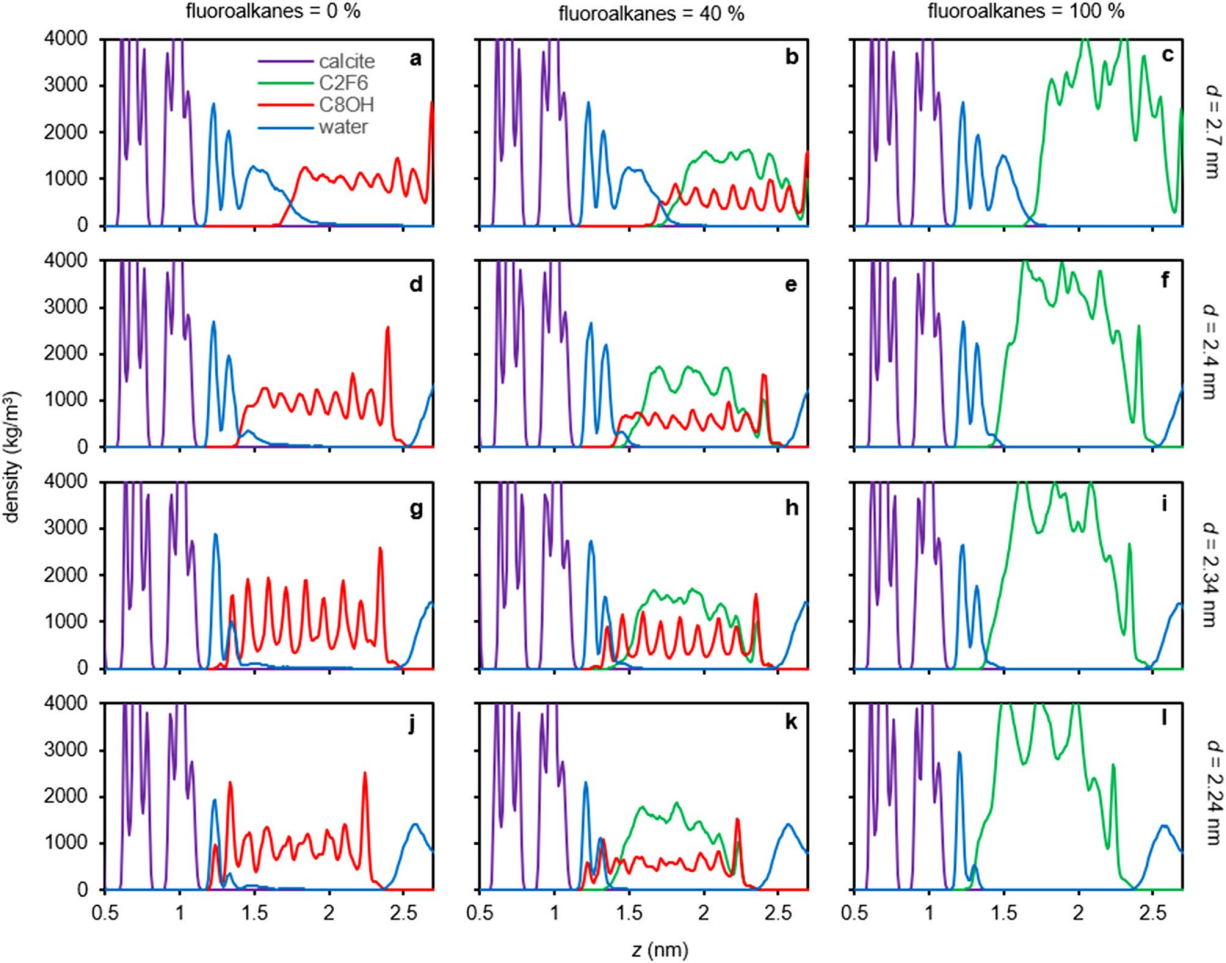
Density distributions for different C8OH-C2F6 SAM-calcite distances with different percentages of fluoroalkane. (**a–c**) *d* = 2.7 nm, (**d–f**) *d* = 2.4 nm, (**g–i**) *d* = 2.34 nm, and (**j–l**) *d* = 2.24 nm with 0% (**a**,**d**,**g**, and **j**), 40% (**b**,**e**,**h**, and **k**), and 100% (**c**,**f**,**i**, and **l**) fluoroalkanes. At *d* = 2.34 nm, the hydroxy groups on C8OH can participate in the second hydration layer (**g** and **h**). At *d* = 2.24 nm, the hydroxy groups on C8OH can participate in both the first and second hydration layers (**j** and **k**). In contrast, the fluoroalkanes cannot participate in the hydration layers (**h**,**i**,**k**, and **l**).

Figure 2a shows the pressure-distance curves for C8OH-C2F6 SAM interactions on calcite surfaces with different percentages of fluoroalkanes (see Methods and Fig. 1c for simulation details and abbreviations for the SAM). As shown, the adhesion pressures decrease with increasing fluoroalkane percentages. Importantly, there are two distinct SAM-calcite distances at *d* = 2.34 and 2.24 nm that show strong adhesions. The molecular origins of these strong adhesions can be best understood in Figs. 3 and 4, which show representative simulation snapshots and density distributions of SAMs with 0%, 40%, and 100% fluoroalkanes at different SAM-calcite distances. For the SAMs relatively far away from the calcite surfaces (Figs. 3a–f and 4a–f; *d* = 2.7 and 2.4 nm), we can see well-defined hydration structures on the calcite surfaces. At the first strong adhesion distance *d* = 2.34 nm, for fluoroalkane-free SAM, the hydroxy groups on the hydrophilic alkanols are mingled comfortably in the second hydration layers on the calcite surfaces (Figs. 3g and 4g). In contrast, for 100% fluoroalkane-SAM, the fluoroalkanes frustrate the hydration layers, instead of participating in them, on the calcite surface (Figs. 3i and 4i). Furthermore, at the second strong adhesion distance *d* = 2.24 nm, for the fluoroalkane-free SAM, the hydroxy groups on the hydrophilic alkanols are mingled comfortably in both the first and second hydration layers on the calcite surfaces (Figs. 3j and 4j). In contrast, for the 100% fluoroalkane-SAM, none of the fluoroalkanes can participate in the calcite hydration layers but only frustrate them (Figs. 3l and 4l). Finally, for the bicomponent SAM with 40% fluoroalkanes, the hydrophilic alkanols partially participate in the calcite hydration layers but the fluoroalkanes don’t (Figs. 3h,k and 4h,k).

### Designing self-assembled monolayers with tunable adhesions on calcite surfaces

Figure 2d shows the potential of mean forces (PMF) for the C8OH-C2F6 SAM interactions on calcite surfaces as integrated from Fig. 2a with different percentages of fluoroalkanes. As shown, the bicomponent SAM become purely repulsive at > 40% mole percentages of fluoroalkanes. As established in the previous section that the fluoroalkanes can perturb the hydration layers on the calcite surfaces and reduce the SAM adhesion, we envisioned that the more fluorinated alkanes should have more pronounced effects. This is clearly observed as in Fig. 2b,c,e,f, which show the pressure-distance curves and PMF for the C8OH-C2F8 and C8OH-C2F10 SAM interactions on calcite surfaces. As shown in Fig. 2e,f, the bicomponent C8OH-C2F8 and C8OH-C2F10 SAM become purely repulsive at > 20% and > 10% fluoroalkanes, respectively.

**Figure 5:**
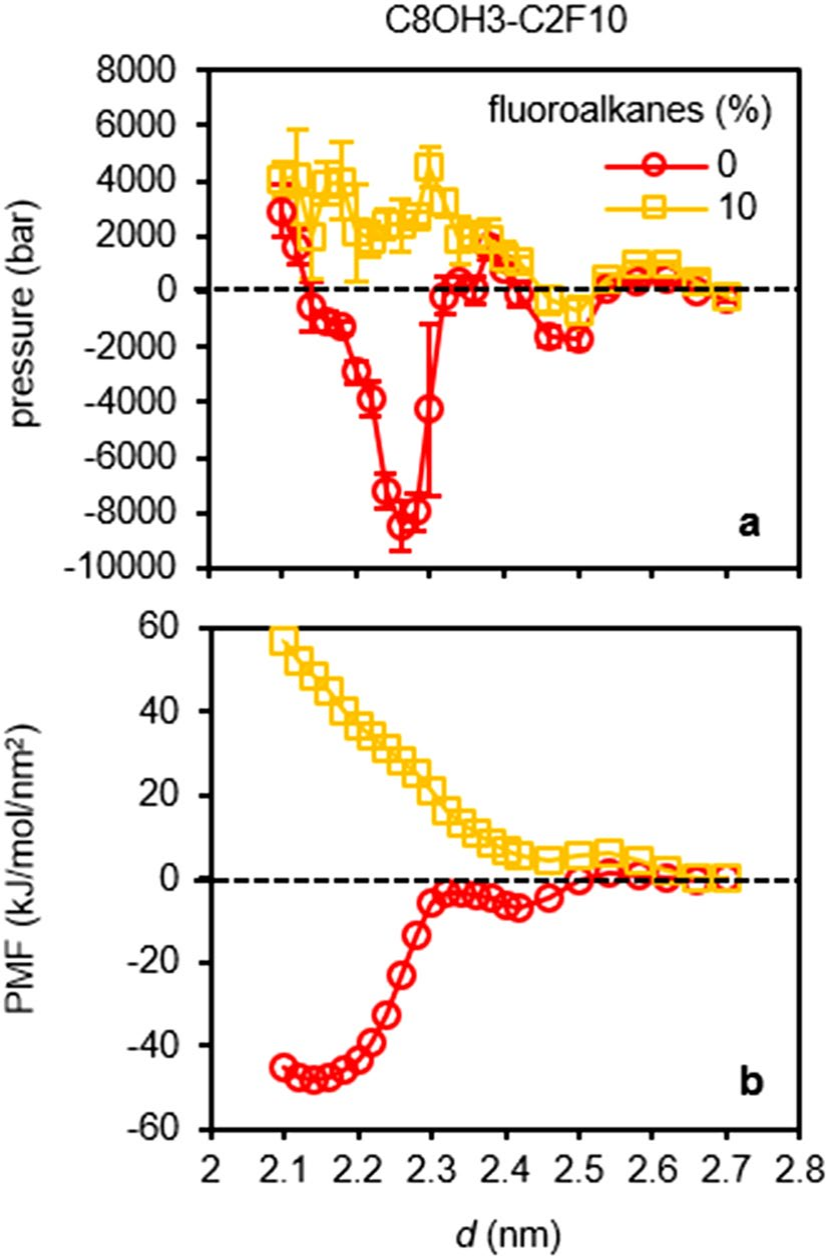
Pressure-distance curves (**a**) and PMF (**b**) for the C8OH3-C2F10 SAM-calcite interactions with different percentages of fluoroalkanes. The pressure-distance curves show a weaker adhesion distance at *d* = 2.5 nm and a very strong adhesion distance at *d* = 2.26 nm. The PMF show a nonstick surface with only 10% C2F10 in a bicomponent SAM.

**Figure 6:**
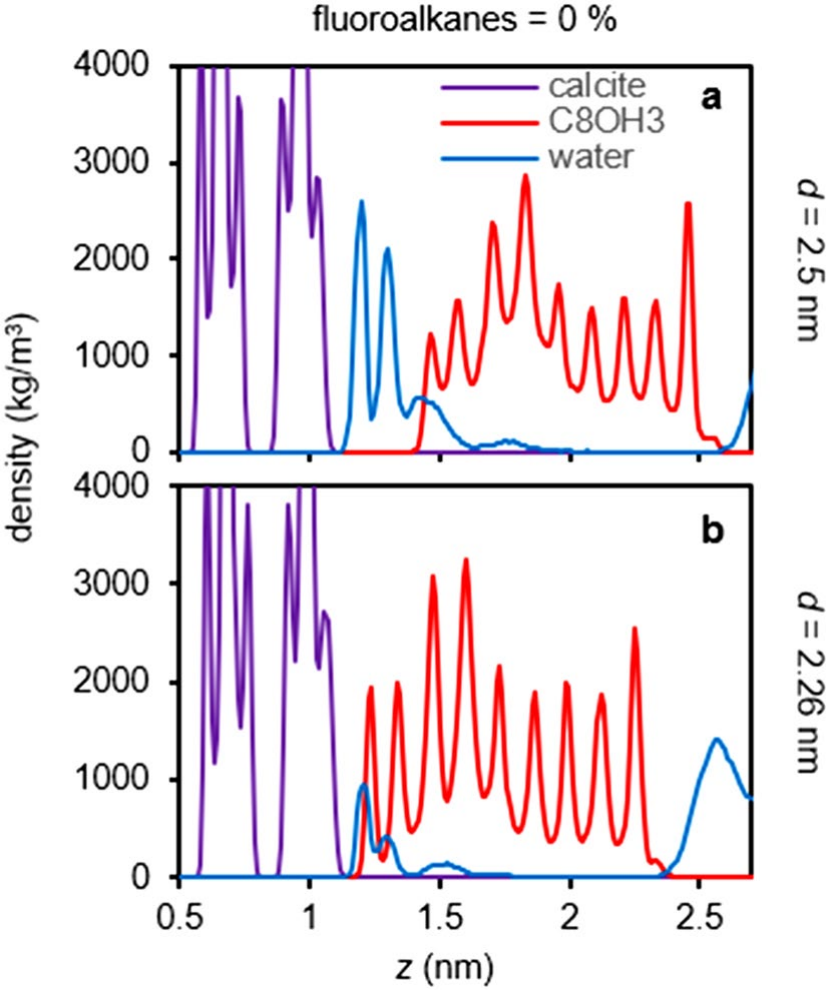
Density distributions for C8OH3 SAM-calcite distances (**a**) *d* = 2.5 nm and (**b**) *d* = 2.26 nm. At *d* = 2.5 nm, the hydroxy groups on C8OH3 can participate in the third hydration layer. At *d* = 2.26 nm, the hydroxy groups on C8OH3 can participate in both the first and second hydration layers.

Finally, we investigated the interactions between calcite surfaces and the SAM consisting of either only a more hydrophilic C8OH3 or bicomponent C8OH3-C2F10 (Fig. 5). For the pure alkanol (C8OH3) SAM (0% fluoroalkane C2F10), the pressure-distance curve in Fig. 5a shows a weak adhesion distance at *d* = 2.5 nm that corresponds to the partitioning of C8OH3 hydroxy groups to the third hydration layer on calcite (Fig. 6a), and a strong adhesion distance at *d* = 2.26 nm that corresponds to the partitioning of C8OH3 hydroxy groups to the first and second hydration layers on calcite (Fig. 6b). Note C8OH3 has multiple hydroxy groups on a single molecule (c.f. Figure 1c) that can concurrently participate in multiple hydration layers on calcites surfaces (Fig. 6b). In contrast, C8OH has only one hydroxy group on a single molecule that can only participate selectively in single hydration layer (Fig. 4g,j). As a result, we observed one continuous strong adhesion when the C8OH3 SAM participated in the first and second hydration layers on calcite (Fig. 5a; *d* = 2.3 to 2.2 nm) instead of the discontinuous two adhesions for C8OH SAM (Fig. 2a–c; *d* = 2.34 to 2.24 nm). Nevertheless, the C8OH3-C2F10 SAM containing 10% fluoroalkane C2F10 becomes purely repulsive (Fig. 5b), which demonstrate the good tunability of the adhesions of the bicomponent SAM on mineral surfaces.

## Discussion

Water-mediated interactions have been intensively studied for many decades^27^. Nevertheless, even though they are ubiquitous in all biological and technological systems, a unifying physical picture has yet to be achieved, and many hydration interactions were considered very surface specific^28–30^. In this work, we study the hydrophilic mineral surfaces, which are a class of very important yet less explored surfaces for hydration interaction. The hydrophilic SAM-mineral interactions could be viewed as nonsymmetric interactions between two hydrophilic surfaces^31^; however, caution should be taken that they may not be understood by merely considering the competition of surface-surface and surface-water interactions, but rather how the highly well-defined water structures on the mineral surfaces (as far as the third hydration layers, see Figs. 5 and 6) may be complied or perturbed, which, we believe, is a unique feature of the hydration forces on mineral surfaces. Finally, we want to stress that the water-mediated interaction is a cooperative process (i.e., nonadditive)^32,33^, as demonstrated here that only 10% fluoroalkane could completely invert the SAM from adhesive to repulsive (c.f. Figs. 2f and 5b). This character has great implications in scaling-up SAM coatings for industrial applications since fluorinated molecules are usually much more expensive compared to the simple hydrophilic alkanols. Reducing the usages of expensive fluorinated molecules while maintaining the desired properties of the SAMs provide immense economic advantages^34,35^.

In summary, in this work using atomistic MD simulations we have studied in detail the water-mediated bicomponent hydrophilic/fluoroalkane SAM interactions on calcite surfaces. We first established that the molecular origin of the strong adhesions of the hydrophilic alkanols on the calcite surfaces were due to the partitioning of the hydroxy groups on the alkanols to the well-defined hydration layers on the calcite surfaces. We then investigated the effects from different types and compositions of fluoroalkanes and found that only 10% of the more fluorinated alkanes (C8OH–C2F10 and C8OH3–C2F10 SAMs) were enough to convert the SAMs to be purely nonstick on calcites. Even though the molecular details put forward in this work have been very helpful in designing the bicomponent surfaces as promising nanoparticle coatings with variable mineral adhesions, further experiments are needed to validate our predictions.

## Methods

The model system consisted of SAMs made with mixtures of hydrophilic alkanols and fluoroalkanes on calcite (1 0 $\overline{1}$ 4) surfaces separated with distances *d* (Fig. 1a). Linear alkanols CH_3_(CH_2_)_7_OH and CH_3_(CH_2_)_4_(CHOH)_2_CH_2_OH (denoted C8OH and C8OH3; Fig. 1c) were used as hydrophilic molecules, and fluoroalkanes CH_3_CH_2_(CF_2_)_5,7,9_CF_3_ (denoted C2F6, C2F8, and C2F10; Fig. 1c) were used to modulate the SAM adhesion on calcite surfaces. The last carbons (on the opposite sides of the hydroxy or fluoro groups) of the bicomponent system were grafted onto a hexagonal lattice with density = 3.8 molecules/nm^2^ that resembled experiment values^36^, and soft restraints were used on the *x* and *y* directions if the molecules exceeded the borders of the outermost grating lattices (Fig. 1b). This design (the position restraint and flat-bottomed position restraint algorithms as implemented in GROMACS software)^37^ ensured the structural integrity of SAM while enabled the proper samplings of the water-mediated SAM-calcite interactions^38^. The calcite, calcite-water, and calcite-SAM molecule interactions were described by the force field of Xiao et al.^39^ in which the hydrophilic and fluoroalkanes were described by the OPLS-AA^40,41^ force field, and the water was treated with the TIP3P^42^ model. The geometric combination rule was used to deduce all pairwise 12–6 Lennard–Jones potentials from atom-wise CaCO_3_, OPLS-AA, and water model parameters except for the H-F interactions, which were further optimized to reproduce experimental alkane and perfluoroalkane mixing properties^43^. This force field combination has been used to study other organic molecule-calcite interactions in solution with good compatibility^44–46^.

Series of MD simulations with different SAM-calcite distances *d* were carried out to obtain the pressure-distance curves under *NPT* condition with *T* = 300 K and *P* = 1 bar. For each *d*, the SAM with area ~ 3 ×  ~ 3 nm^2^ was placed at the designed height above an extended calcite slab perpendicular to the *z* axis in a simulation box with initial box size 5 × 5 × 5 nm^3^ and periodic boundary conditions in three dimensions (Fig. 1a). The simulation boxes with predefined SAM and calcite surfaces were then solvated by 2400 water molecules. For each pressure-distance curve, we performed simulations with *d* = 2.70 to 2.42 nm with 0.04 nm increments followed by *d* = 2.40 to 2.10 nm with 0.02 nm increments. The simulation times for the MD runs were 40 ns for *d* > 2.40 nm and 100 ns for *d* ≤ 2.40 nm, with data analysis performed for the last 20 ns. Statistical uncertainties for the force measurements at each *d* were acquired by performing 3 independent simulations with randomized initial configurations of the SAMs.

At each SAM-calcite distance *d*, the pressures on SAM were measured from MD trajectories. At each MD time step, we first summed the total forces exerted on all the atoms that construct the SAM, and then calculated by dividing the sum of total forces on SAM by the projected area of SAM on the *x–y* plane to give the pressures. Finally, the potential of mean forces (PMF) were calculated by ${\text{PMF}}\left( d \right) = \mathop \int \limits_{d}^{\infty } {\Pi }\left( {d^{{\prime }} } \right)dd^{{\prime }}$, where Π(*d*) were the pressures measured at each *d* as described above (i.e., pressure-distance curves) and we used the max distance *d* = 2.7 nm as the reference state^19,33^.

All simulations were carried out using GROMACS v2018.2^37^. Electrostatic interactions were calculated using the particle-mesh Ewald summation, with a real-space cutoff of 1 nm, a grid spacing of 0.16 nm, and fourth-order interpolation. The van der Waals and neighbor-list cutoffs were both set to 1 nm. We used velocity rescaling temperature coupling with a time constant of 0.5 ps and Berendsen semi-isotropic pressure coupling with a time constant of 5 ps. The simulation time step was set to 2 fs. The bottom 2 layers of the calcite molecules were restrained (using GROMACS position restraint algorithm)^37^ in order to support the surface, while the top 2 layers of the calcite molecules facing the SAM could freely move^45,46^. All bonds were constrained using the LINCS algorithm^47^ except for water molecules, which were constrained using the SETTLE algorithm^48^.

## Data Availability

The data sets generated and analyzed during the current study are available from the corresponding author on reasonable request.

## References

[1] Kim K, Fisher JP (2007). Nanoparticle technology in bone tissue engineering. J. Drug Targeting.

[2] Tautzenberger A, Kovtun A, Ignatius A (2012). Nanoparticles and their potential for application in bone. Int. J. Nanomedicine.

[3] Cheng H (2017). Development of nanomaterials for bone-targeted drug delivery. Drug Discovery Today.

[4] Krohn-Grimberghe, M. *et al.* Nanoparticle-encapsulated siRNAs for gene silencing in the haematopoietic stem-cell niche. *Nat. Biomed. Eng.*, 1–14 (2020). 10.1038/s41551-020-00623-7 PMC7655681 33020600

[5] Balakumaran A (2010). Superparamagnetic iron oxide nanoparticles labeling of bone marrow stromal (mesenchymal) cells does not affect their “stemness”. PLoS ONE.

[6] Kim J (2017). Use of nanoparticle contrast agents for cell tracking with computed tomography. Bioconjugate Chem..

[7] Navrotsky A (2004). Energetic clues to pathways to biomineralization: Precursors, clusters, and nanoparticles. Proc. Natl. Acad. Sci. USA.

[8] Chen Y (2019). Biomineralization forming process and bio-inspired nanomaterials for biomedical application: a review. Minerals.

[9] Huang L-H, Sun X-Y, Ouyang J-M (2019). Shape-dependent toxicity and mineralization of hydroxyapatite nanoparticles in A7R5 aortic smooth muscle cells. Sci. Rep..

[10] Zhang W-X (2003). Nanoscale iron particles for environmental remediation: an overview. J. Nanopart. Res..

[11] Berlin JM (2011). Engineered nanoparticles for hydrocarbon detection in oil-field rocks. Energy Environ. Sci..

[12] Worthen AJ (2013). Nanoparticle-stabilized carbon dioxide-in-water foams with fine texture. J. Colloid Interface Sci..

[13] Zhang H, Nikolov A, Wasan D (2014). Enhanced oil recovery (EOR) using nanoparticle dispersions: underlying mechanism and imbibition experiments. Energy Fuels.

[14] Ko S, Huh C (2019). Use of nanoparticles for oil production applications. J. Pet. Sci. Eng..

[15] Gizzatov A (2019). Nanofluid of petroleum sulfonate nanocapsules for enhanced oil recovery in high temperature and salinity reservoirs. Energy Fuels.

[16] Chen H, Gizzatov A, Abdel-Fattah AI (2020). Molecular assembly of surfactant mixtures in oil-swollen micelles: implications for high salinity colloidal stability. J. Phys. Chem. B.

[17] Singh N, Jenkins GJ, Asadi R, Doak SH (2010). Potential toxicity of superparamagnetic iron oxide nanoparticles (SPION). Nano Rev..

[18] Chen H, Cox JR, Panagiotopoulos AZ (2016). Force fields for carbohydrate–divalent cation interactions. J. Phys. Chem. B.

[19] Chen H, Cox JR, Ow H, Shi R, Panagiotopoulos AZ (2016). Hydration repulsion between carbohydrate surfaces mediated by temperature and specific ions. Sci. Rep..

[20] Liu Q, Zhang Y, Laskowski J (2000). The adsorption of polysaccharides onto mineral surfaces: an acid/base interaction. Int. J. Miner. Process..

[21] Laskowski, J., Liu, Q. & O'connor, C. Current understanding of the mechanism of polysaccharide adsorption at the mineral/aqueous solution interface. *Int. J. Miner. Process.* **84**, 59–68 (2007).

[22] Wise ER (2007). The organic-mineral interface in bone is predominantly polysaccharide. Chem. Mater..

[23] Witzler M (2019). Polysaccharide-based systems for targeted stem cell differentiation and bone regeneration. Biomolecules.

[24] Chuang Y-J (2016). Ultra-sensitive in-situ detection of near-infrared persistent luminescent tracer nanoagents in crude oil-water mixtures. Sci. Rep..

[25] Chang S, Eichmann SL, Huang T-YS, Yun W, Wang W (2017). Controlled design and fabrication of SERS–SEF multifunctional nanoparticles for nanoprobe applications: morphology-dependent SERS phenomena. J. Phys. Chem. C.

[26] Chen H, Shi R, Ow H (2019). Predicting stability constants for terbium (III) complexes with dipicolinic acid and 4-substituted dipicolinic acid analogues using density functional theory. ACS Omega.

[27] Israelachvili J, Wennerstrom H (1996). Role of hydration and water structure in biological and colloidal interactions. Nature.

[28] Parsegian V, Zemb T (2011). Hydration forces: observations, explanations, expectations, questions. Curr. Opin. Colloid Interface Sci..

[29] Schneck E, Sedlmeier F, Netz RR (2012). Hydration repulsion between biomembranes results from an interplay of dehydration and depolarization. Proc. Natl. Acad. Sci. USA.

[30] Kanduč M, Netz RR (2015). From hydration repulsion to dry adhesion between asymmetric hydrophilic and hydrophobic surfaces. Proc. Natl. Acad. Sci. USA.

[31] Kanduč M, Schlaich A, Schneck E, Netz RR (2016). Water-mediated interactions between hydrophilic and hydrophobic surfaces. Langmuir.

[32] Giovambattista N, Debenedetti PG, Rossky PJ (2007). Hydration behavior under confinement by nanoscale surfaces with patterned hydrophobicity and hydrophilicity. J. Phys. Chem. C.

[33] Hua L, Zangi R, Berne B (2009). Hydrophobic interactions and dewetting between plates with hydrophobic and hydrophilic domains. J. Phys. Chem. C.

[34] He Y (2020). Brine-soluble zwitterionic copolymers with tunable adsorption on rocks. ACS Appl. Mater. Interfaces.

[35] Kawelah M (2020). Dynamic adsorption of functionalized zwitterionic copolymers on carbonate surfaces under extreme reservoir conditions. Energy Fuels.

[36] Roscioni, O. M., Muccioli, L., Mityashin, A., Cornil, J. R. M. & Zannoni, C. Structural characterization of alkylsilane and fluoroalkylsilane self-assembled monolayers on SiO2 by molecular dynamics simulations. *J. Phys. Chem. C* **120**, 14652–14662 (2016).

[37] Abraham MJ (2015). GROMACS: High performance molecular simulations through multi-level parallelism from laptops to supercomputers. SoftwareX.

[38] Dallin BC, Yeon H, Ostwalt AR, Abbott NL, Van Lehn RC (2019). Molecular order affects interfacial water structure and temperature-dependent hydrophobic interactions between nonpolar self-assembled monolayers. Langmuir.

[39] Xiao S, Edwards SA, Gräter F (2011). A new transferable forcefield for simulating the mechanics of CaCO_3_ crystals. J. Phys. Chem. C.

[40] Jorgensen WL, Maxwell DS, Tirado-Rives J (1996). Development and testing of the OPLS all-atom force field on conformational energetics and properties of organic liquids. J. Am. Chem. Soc..

[41] Watkins EK, Jorgensen WL (2001). Perfluoroalkanes: Conformational analysis and liquid-state properties from ab initio and Monte Carlo calculations. J. Phys. Chem. A.

[42] Jorgensen WL, Chandrasekhar J, Madura JD, Impey RW, Klein ML (1983). Comparison of simple potential functions for simulating liquid water. J. Chem. Phys..

[43] Song W, Rossky PJ, Maroncelli M (2003). Modeling alkane+perfluoroalkane interactions using all-atom potentials: failure of the usual combining rules. J. Chem. Phys..

[44] Chen H, Panagiotopoulos AZ, Giannelis EP (2015). Atomistic molecular dynamics simulations of carbohydrate–calcite interactions in concentrated brine. Langmuir.

[45] Chen H, Eichmann SL, Burnham NA (2019). Understanding calcium-mediated adhesion of nanomaterials in reservoir fluids by insights from molecular dynamics simulations. Sci. Rep..

[46] Chen H, Eichmann SL, Burnham NA (2020). Specific ion effects at calcite surface defects impact nanomaterial adhesion. J. Phys. Chem. C.

[47] Hess B (2008). P-LINCS: a parallel linear constraint solver for molecular simulation. J. Chem. Theory Comput..

[48] Miyamoto S, Kollman PA (1992). SETTLE: an analytical version of the SHAKE and RATTLE algorithm for rigid water models. J. Comput. Chem..

